# Barriers to Creutzfeldt-Jakob Disease Autopsies, California

**DOI:** 10.3201/eid1009.040066

**Published:** 2004-09

**Authors:** Janice K. Louie, Shilpa S. Gavali, Ermias D. Belay, Rosalie Trevejo, Lucinda H. Hammond, Lawrence B. Schonberger, Duc J. Vugia

**Affiliations:** * California Emerging Infections Program, Richmond, California, USA;; † California Department of Health Services, Berkeley, California, USA;; ‡ Centers for Disease Control and Prevention, Atlanta, Georgia, USA

**Keywords:** Creutzfeldt-Jakob Disease, CJD, variant, transmissible spongiform encephalopathy, TSE, prion, surveillance, autopsy, dispatch

## Abstract

Creutzfeldt-Jakob disease (CJD) surveillance relies on autopsy and neuropathologic evaluation. The 1990–2000 CJD autopsy rate in California was 21%. Most neurologists were comfortable diagnosing CJD (83%), but few pathologists felt comfortable diagnosing CJD (35%) or performing autopsy (29%). Addressing obstacles to autopsy is necessary to improve CJD surveillance.

Transmissible spongiform encephalopathies (TSEs) are rare, progressively fatal, neurodegenerative illnesses. Human TSEs include classic Creutzfeldt-Jakob disease (CJD) and the recently described variant CJD associated with eating bovine spongiform encephalopathy–infected cattle products in Europe (*1*). The recent identification of bovine spongiform encephalopathy in the United States underscores the importance of maintaining enhanced surveillance to monitor for the possible occurrence of variant CJD in this country (*2**,**3*).

In California, CJD is not reportable. Since 1999, the California CJD Surveillance Project of the California Emerging Infections Program, a collaboration of the California Department of Health Services and the U.S. Centers for Disease Control and Prevention, has conducted enhanced surveillance for classic and variant CJD. Methods include review of state mortality data and follow-up investigation of CJD-related deaths that occur in persons <55 years of age, since >98% of cases of variant CJD in the United Kingdom have occurred in this age group. As part of this enhanced surveillance, medical records for 33 deceased California residents <55 years old from 1996 through 2003 have been investigated with criteria for CJD developed by the World Health Organization and Centers for Disease Control and Prevention; none met the criteria for variant CJD.

Currently, pathologic review of brain tissue obtained by biopsy or autopsy is the only means of confirming a diagnosis of CJD. Autopsy remains the preferred method for obtaining tissue, as brain biopsy can result in serious complications (e.g., brain hemorrhage or abscess formation) and may not yield adequate amounts of tissue for analysis. The main role of brain biopsy is to exclude other, potentially treatable conditions (*4*).

In this article, we describe results from analysis of California mortality data from 1990 through 2000. We also summarize responses generated from a statewide survey of neurologists and pathologists regarding the challenges to diagnosing CJD and variant CJD, including obtaining autopsy in suspected cases.

## The Study

Data from the 1990–2000 Death Public Use File (underlying cause of death only) and 1990–1999 Multiple Cause-of-Death Data (underlying or contributing causes of death) were obtained from the Center for Health Statistics, California Department of Health Services (*5*). Deaths among California residents with an International Classification of Diseases, 9th Revision, code 046.1 or 10th Revision, code A81.0 listed anywhere on the death record were included in our analysis. Both data files included report of autopsy as a variable, with the exception of the Multiple Cause-of-Death Data for 1997 to 1999, when autopsy performance was not recorded. Statistical analysis was performed by using SAS software (SAS Institute, Cary, NC).

From July to December 2002, questionnaires regarding experience with diagnosing CJD were sent to 1,241 California neurologists identified as members of the American Academy of Neurology and 574 pathologists identified as members of the California Society of Pathologists and the American Association of Neuropathologists. Approval was obtained from the Committee for the Protection of Human Subjects of the State of California.

Review of mortality data identified 263 CJD-related deaths in California from 1990 through 2000. Of these, 244 were identified from the 1990–1999 Multiple Cause-of-Death Data, and an additional 19 deaths were identified from the 1990–2000 Death Public Use File. A total of 42 (16%) cases identified by the Multiple Cause-of-Death Data were not detected in the Death Public Use File. Overall, 26 (10%) of the 263 CJD-related deaths were in persons <55 years of age. Only two deaths occurred in persons <30 years of age. The overall autopsy rate, which for 1997 to 2000 only includes autopsies performed on persons for whom CJD was recorded as the underlying cause of death, was 53 (21%) of 251 persons: 11 (44%) of 25 persons <55 years of age, and 42 (19%) of 226 persons >55 years of age. For two deaths, autopsy performance was not recorded.

Of 1,241 questionnaires mailed to neurologists, 428 (34%) were completed, including 310 (25%) from respondents involved in patient care. Responses regarding the neurologists' experience with diagnosing CJD and performing autopsy are summarized in Table 1 and Table 2. Most neurologists (83%, 255/307) felt comfortable clinically recognizing classic CJD. More than one third (36%, 74/207) had not considered arranging for autopsy in their CJD patients, although most reported access to histopathologic services (75%, 223/297). The most commonly cited barrier to obtaining autopsy was family reluctance to give consent (79%, 192/242).

**Table 1:** Knowledge and experience of California neurologists, pathologists, and neuropathologists in diagnosing Creutzfeldt-Jakob disease (CJD)

Characteristic	Neurologists n/N (%)	Pathologists n/N (%)	Neuropathologists n/N (%)
Have evaluated a case of CJD	212/310 (68)	56/259 (22)	18/33 (55)
Median no. (range) of CJD cases evaluated	3 (0–30)	2 (0–30)	10 (0–50)
Type of practice			
Private practice/private hospital	144/308 (47)	122/278 (44)	8/33 (25)
Outpatient HMO^a^/managed care	55/308 (18)	–	–
Community hospital/clinic	1/308 (<1)	68/278 (24)	4/33 (12)
University affiliated	82/308 (27)	37/278 (13)	10/33 (30)
Veterans hospital	13/308 (4)	3/278 (1)	1/33 (3)
County medical examiner or coroner	–	7/278 (3)	2/33 (6)
Other	15/308 (5)	41/278 (15)	5/33 (15)
Can recognize the clinical or pathologic features of classic CJD	255/307 (83)	96/273 (35)	25/28 (89)
Can recognize the clinical or pathologic features of variant CJD	120/305 (39)	40/274 (15)	18/28 (64)
Have not considered arranging for an autopsy for CJD patients under their care	74/207 (36)	–	–
Pathology group available at facility to perform autopsy on suspect CJD cases	223/297 (75)	74/259 (29)	17/28 (61)
Pathology group available at facility to confirm diagnosis of suspect CJD with histopathologic analysis	223/297 (75)	91/254 (36)	18/27 (67)

^a^HMO, health maintenance organization.

**Table 2:** Perceptions of California neurologists, pathologists, and neuropathologists regarding performance of autopsy in Creutzfeldt-Jakob disease (CJD)

Characteristic	Neurologists n/N (%)	Pathologists n/N (%)	Neuropathologists n/N (%)
Important reasons to obtain autopsy for CJD patients			
Autopsy is needed to confirm CJD diagnosis	–	92/197 (47)	11/21 (52)
Autopsy is needed to rule out variant CJD or other TSE^a^ forms	168/231 (73)	87/193 (45)	12/20 (60)
Barriers to performing autopsy and histopathologic analysis for CJD			
Clinicians do not feel autopsy is required for diagnosis	94/221 (43)	72/198 (36)	7/21 (33)
Facilities not able/willing to perform autopsies on CJD patients	75/234 (32)	111/210 (53)	8/22 (36)
Families are reluctant to give consent for autopsy	192/242 (79)	57/202 (28)	6/22 (27)
Cost of autopsy is a concern to patient's family	113/234 (48)	34/202 (17)	8/20 (40)
Cost of autopsy is a concern to hospital/institution	78/234 (34)	40/199 (20)	8/21 (38)
Infection control is a concern regarding autopsy	102/235 (44)	143/185 (77)	9/11 (82)
Facilities are inadequate to perform autopsy	–	24/185 (13)	5/11 (45)
Infection control is a concern regarding histopathologic evaluation	–	62/111 (56)	4/8 (50)
No available pathologists experienced in recognizing histopathologic features of CJD	–	69/111 (62)	1/8 (13)
^a^TSE, transmissible spongiform encephalopathy.			

Of 574 questionnaires mailed to pathologists, 284 (49%) were completed. Table 1 and Table 2 summarize the responses. Thirty-five percent (96/273) and 15% (40/274) of pathologists were comfortable recognizing the neuropathologic features of classic CJD and variant CJD, respectively. Infection control concerns (77%, 143/185), lack of experience (62%, 69/111), and institutional limitations (53%, 111/210) were cited as major obstacles to autopsy performance, and less than half of respondents reported that confirming the diagnosis of CJD (47%, 92/197) or ruling out variant CJD (45%, 87/193) was an important reason to pursue autopsy.

## Conclusions

Our analysis suggests that autopsy rates for CJD in California are low. The results of our surveys, which attempted to discern the reasons for this low rate, imply that both neurologists and pathologists have similar perceptions of the value of obtaining histopathologic evaluation for CJD but for different reasons. Most neurologists appeared to be comfortable clinically diagnosing CJD, with more than one third reporting they had never considered pursuing autopsy for CJD cases. In contrast, pathologists appeared to be less comfortable making a histopathologic diagnosis, indicating that autopsy performance was limited by infection control concerns, lack of experience with CJD cases, and institutional restrictions.

Our results have some limitations. Approximately 10% of CJD cases may have atypical signs and symptoms that can obscure the diagnosis. To the extent that these cases are misdiagnosed and not autopsied, they could contribute to overestimation of the autopsy rate. On the other hand, death certificate analysis can be an insensitive indicator of the true rate of autopsy, and autopsy performance information was unavailable for 1997 to 2000 from the Multiple Cause-of-Death Data. Both factors could lead to possible underestimation of the true autopsy rate. Given that some CJD cases will have had confirmatory brain biopsy or strongly suggestive clinical features and diagnostic studies, the autopsy rates cited may apply mostly to patients for whom a satisfactory antemortem diagnosis could not be made. Interpreting survey results is limited by the low response rate; neurologists and pathologists who are experienced in diagnosing CJD may be more likely to respond, which would introduce bias.

The public health benefits of performing autopsy on patients with suspected CJD should not be underestimated. Autopsy and histopathologic analysis remain important ways to confirm a diagnosis of CJD and help define the usual occurrence of subtypes of classic CJD, thereby facilitating the recognition of emerging TSEs (*1**,**6**,**7*). Autopsy rates for nonforensic deaths have declined dramatically during the past 40 years, with national hospital rates currently <5%, possibly resulting in missed diagnoses of the actual cause of death in 8% to 25% of cases (*8**–**11*). The reasons for the decline are multifaceted and include escalating cost of autopsy borne by hospitals and county medical examiners, lack of direct reimbursement, fear of litigation, and increasing reliance on modern technology to determine a diagnosis antemortem (*10*).

Our survey results suggest that infection control concerns play a role in low autopsy rates for CJD, whether because of fears about the risk of acquiring CJD from handling contaminated tissue or because of liability considerations at the institutional level. More realistically, brain autopsy can be performed safely as long as CJD-specific infection control guidelines are strictly followed (*12**–**13*). Nonetheless, concerns about potentially acquiring CJD through autopsy procedures should be acknowledged and recognized as an opportunity to address proper infection control techniques.

Enhancing surveillance for variant CJD and other emerging prion diseases will require educating neurologists and pathologists, addressing the perceived obstacles to obtaining autopsy, and encouraging the use of available resources that provide expertise and technical assistance in evaluating CJD. For example, brain tissue can be submitted to the National Prion Disease Pathology Surveillance Center (NPDPSC) in Cleveland, Ohio, for free state-of-the-art diagnostic testing (*14*). The availability of a national center of expertise may facilitate obtaining tissue evaluation; since the inception of NPDPSC, the number of referrals to the facility has more than doubled, from 104 in 1997 to 265 in 2002, and the number of TSE cases confirmed from those referrals increased from 60 in 1997 to 151 in 2002 (*14*). Regional academic institutions, such as the University of California, San Francisco, Memory and Aging Center, can also provide expertise and assistance with diagnostic testing. Such resources are vital to maintaining vigilance for cases of CJD and potentially emerging human TSEs, such as variant CJD or possibly a human form of chronic wasting disease in the United States.
